# A Pilot Study: the Development of a Facility-Associated Microbiome and Its Association with the Presence of *Listeria* Spp. in One Small Meat Processing Facility

**DOI:** 10.1128/spectrum.02045-22

**Published:** 2022-08-18

**Authors:** Aeriel D. Belk, A. Nathan Frazier, Luke K. Fuerniss, Robert Delmore, Keith Belk, Brad Borlee, Ifigenia Geornaras, Jennifer N. Martin, Jessica L. Metcalf

**Affiliations:** a Department of Animal Sciences, Colorado State University, Fort Collins, Colorado, USA; b Joint Institute for Food Safety and Applied Microbiology, University of Maryland, College Park, Maryland, USA; c Department of Microbiology, Immunology, and Pathology, Colorado State University, Fort Collins, Colorado, USA; d CIFAR Azrieli Global Scholars Program, CIFAR, Toronto, Canada; University of Turin

**Keywords:** *Listeria*, *Listeria monocytogenes*, built environment, food safety, meat processing, microbiome

## Abstract

Microbial communities which persist in food processing facilities may have a detrimental impact on food safety and spoilage. In meat processing, Listeria monocytogenes is an organism of concern due to its ability to cause significant human illnesses and persist in refrigerated environments. The microbial ecology of *Listeria* spp. in small meat processing facilities has not been well characterized. Therefore, we collected samples from a newly constructed meat processing facility as an opportunity to investigate several research objectives: (i) to determine whether a stable, consistent microbiome develops in a small meat processing facility during the first 18 months of operation, (ii) to evaluate the environmental factors that drive microbial community formation, and (iii) to elucidate the relationship between microbial communities and the presence of *Listeria* species. We evaluated microbiomes using 16S rRNA gene sequencing and *Listeria* presence using quantitative PCR. We demonstrated that microbial communities differentiate by the functional room type, which is representative of several environmental differences such as temperature, sources of microbes, and activity. Temperature was an especially important factor; in rooms with low temperatures, communities were dominated by psychotrophs, especially Pseudomonas, while warmer rooms supported greater diversity. A stable core community formed in facility drains, indicating that mechanisms which cause persistence are present in the communities. The overall presence of *Listeria* in the facility was low but could be tied to specific organisms within a room, and the species of *Listeria* could be stratified by room function.

**IMPORTANCE** This study provides critical knowledge to improve meat safety and quality from small meat processing facilities. Principally, it demonstrates the importance of facility design and room condition to the development of important microbial communities; temperature, sanitation regimen, and physical barriers all influence the ability of microorganisms to join the stable core community. It also demonstrates a relationship between the microbial community and *Listeria* presence in the facility, showing the importance of managing facility sanitation plans for not only pathogens, but also the general facility microbiome.

## INTRODUCTION

The growing field of built environment microbiome studies has allowed researchers to focus on the unique relationship between humans and the environments they occupy. Generally, the spaces investigated in these studies are facilities in which humans spend a majority of their time and in which they are the major occupant; for example, homes (1–4), school and university buildings (5–7), hospitals (8), and athletic facilities (9, 10). In nearly every case, the microbial composition of the built environment has been shown to reflect that of the primary occupants, even in cases with consistent cleaning and sanitation practices. Not only this, but the transition of the microbial community to reflect the occupants occurs very rapidly, often in less than a day (4, 8). However, humans are not the primary occupant of many under-studied built environments, although they can be negatively impacted by the microbial communities found in their spaces. Specifically, the microbial communities in food and beverage production facilities may have important implications on the safety and quality of commercial food products. In this case, the food products, and not the humans, are the primary occupants of the facilities.

Microorganisms are generally considered the enemies of wholesome food production systems. Some microbes which enter a food production facility, especially those for foods of animal origin, are pathogens of concern. Notably, the presence or persistence of Listeria monocytogenes in facilities poses a significant food safety risk because its presence on equipment surfaces or in the environment can potentially result in contamination of products considered ready-to-eat, which will not be cooked prior to consumption. Listeriosis, the illness associated with L. monocytogenes infection, has the highest mortality rate among foodborne illnesses; this organism is responsible for 1,662 cases and 19% of deaths related to foodborne illness in the United States each year (11). Importantly, L. monocytogenes can grow under refrigerated conditions, such as those maintained in food processing facilities. There are two species of *Listeria* generally isolated from food processing facilities: L. monocytogenes and L. innocua. Both species are able to contaminate food products, but in general only L. monocytogenes is pathogenic to humans (12). To reduce risk of contamination from pathogens such as L. monocytogenes, food processing facilities are required to have testing and control plans (13). However, the majority of organisms present in the food production environment are unlikely to cause human illness, but instead impact the microbiological quality of fresh meat products by their involvement in spoilage, especially organisms such as Pseudomonas spp. and lactic acid bacteria. Moreover, there is evidence that these organisms can be transferred from the environment to the food products, demonstrating the critical importance of managing the built environment microbiome (14, 15). As a result, advances have been made by food production industries in an effort to reduce the presence of microbes in their facilities, such as the introduction of rigorous cleaning and sanitation regimes and the use of facility design to prevent cross-contamination between processing steps. Despite these efforts, resident microbial communities, sometimes associated with the presence of *Listeria* spp., have been identified in brewing (15), fruit processing (16), and meat processing (14, 17–19) facilities. Though a general picture of these communities is forming through the published literature, there is still a major knowledge gap surrounding the sources of these microbes, how the communities assemble within the facility, and the relationship between the microbial community and the establishment and persistence of pathogens such as L. monocytogenes.

We investigated the establishment of surface microbial communities in a new, industry-scale small meat production facility housed in the Global Food Innovation Center in honor of Gary and Kay Smith in the Animal Sciences department at Colorado State University. We addressed several objectives: (i) determine whether a stable, consistent microbiome develops in a small meat processing facility during the first 18 months of operation, (ii) evaluate the environmental factors which drive microbial community formation, and (iii) elucidate the relationship between microbial communities and the presence of *Listeria* species.

## RESULTS AND DISCUSSION

### Overview.

**FIG 1:**
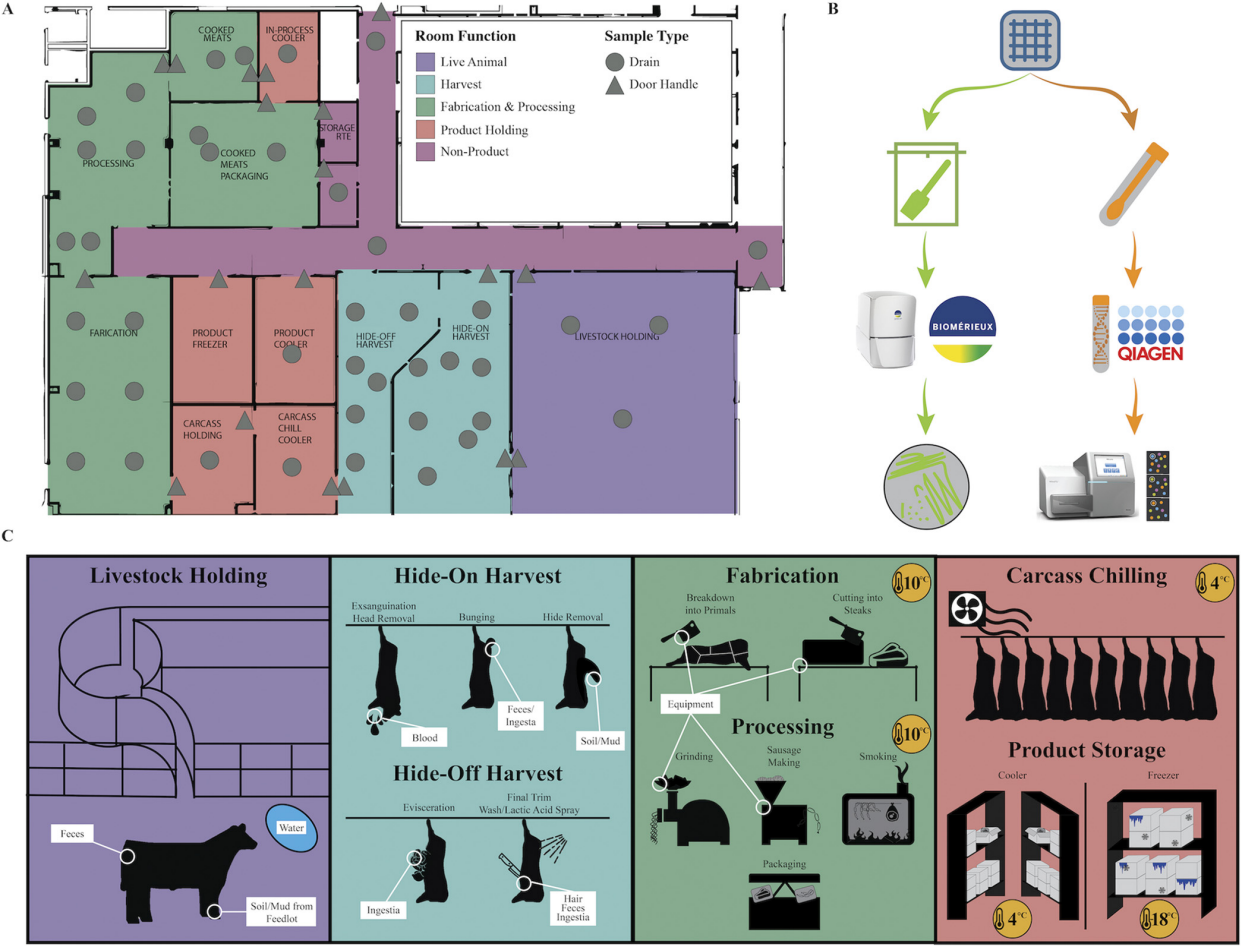
Global Food Innovation Center (GFIC) facility layout and experimental design. Rooms within the facility are categorized by general function. (A) Facility map labeled with sampling points. Sampling sites are identified by their location within the facility by either circles (drains) or triangles (door handles). (B) Flow chart representing the sample processing protocol for this experiment. Sponge samples were collected to determine the presence of *Listeria* spp. and swabs were used to collect microbial DNA for amplicon sequencing. (C) Representation of activities that occur within given processing spaces and potential mechanisms for microbial transfer in these spaces.

To investigate the changes in the microbial communities within a newly constructed meat processing facility in the Global Food Innovation Center (GFIC; Fort Collins, CO), we collected samples monthly from drains and door handles throughout the facility from January 2019 until August 2020. Fig. 1 provides an overview of the experimental design and sampling scheme for the experiment. Specifically, swab samples were collected and used for 16S rRNA gene sequencing to profile the microbiome and sponge samples were collected to evaluate *Listeria* spp. prevalence. This experimental period spanned from post-construction cleaning (pre-opening) through 18 months of operation.

### Sequencing results.

The microbiome was evaluated using sequencing of the V4 region of the 16S rRNA gene following Earth Microbiome Project (EMP) protocols. In total, 1,009 samples were sequenced for this study, including 39 negative and no-template controls and 4 mock communities. Sequencing resulted in a total of 34,332,385 paired-end reads. After denoising, quality-filtering, read-joining, and chimera removal with the DADA2 plugin, the data set contained 31,676 distinct amplicon sequence variants (ASVs) in a total frequency of 25,110,424 ASVs (range: 1 to 152,682 ASV/sample, mean: 24,886 ASV/sample). These samples were filtered to remove ASVs which were assigned to chloroplasts and mitochondria after taxonomic analysis, resulting in 29,485 ASVs.

Negative controls were evaluated based on the number of reads present and were all determined to be low counts (0 to 6,533 reads/sample, mean = 588), indicating that these data could be considered uncontaminated. Additionally, the rarefaction level used in diversity analysis for this study was well above the threshold of the highest negative control, giving us confidence in our biological samples. Mock communities with 13 species were sequenced for inclusion as positive controls. The taxonomic profiles of these communities were compared with the expected composition based on manufacturer reports. The mock communities resulted in the expected community with no unexpected taxa, indicating expected sequencing quality and no major contamination (Fig. S1 in Supplemental File 1). All negative- and positive-control samples were removed from the data set before further analysis.

### Environmental conditions impacting microbial ecology.

Within the meat processing facility, the microbial communities are differentiated by functional room types. These functional types are representative of numerous environmental differences, including the room temperature, the products and ingredients present in the space, the activity occurring in the space, and the potential sources of microbes, all of which have been previously shown to influence the built environment microbiome (1–4, 7, 8, 20, 21). In the GFIC, these spaces are physically and functionally separated, which further isolates the microbes within a space and prevents microbial movement or transfer between these functional room types (Fig. S2). This environmental restriction leads to microbes being contained in the particular space, which increases the importance of environmental selection (8, 15).

**FIG 2:**
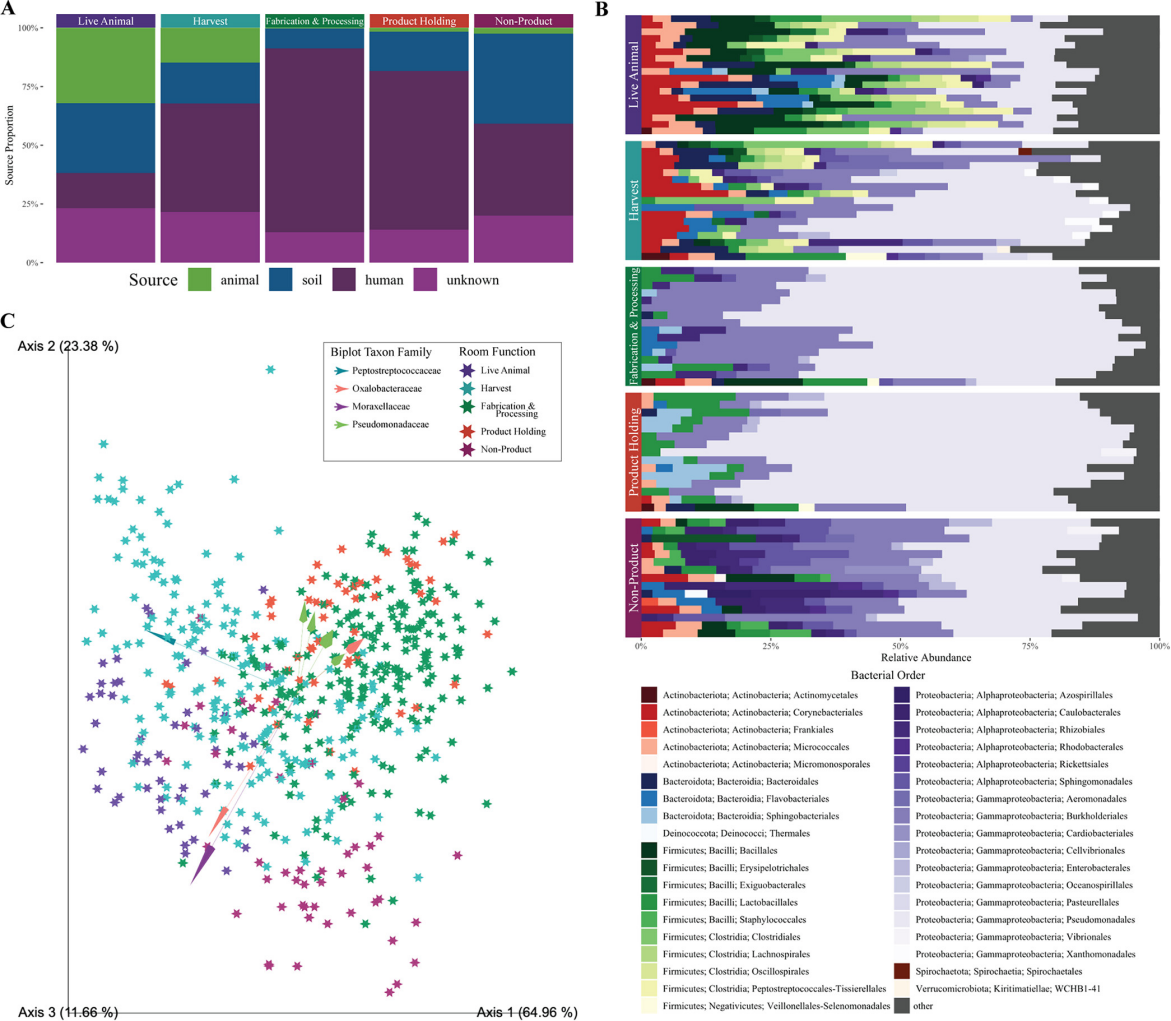
Microbial communities are differentiated by functional room types. (A) Proportion of the microbial community within a room-function group contributed by each potential microbe source. (B) Taxonomy associated with each room-function group. Colors represent different microbial orders, and within a room-function facet, the bars are ordered by sampling event from the bottom (line 1) to the top (line 15). (C) Principal coordinates plot, using the Robust Aitchison distance, of the microbial communities, showing separation by room functional types. Arrows represent amplicon sequence variants (ASVs) that drive separation of clusters.

Microbial community composition in the GFIC spaces is influenced by the distinct sources that interact with the environment. It is well established that the occupants of a built environment have a significant impact on the microbial community (3, 4, 8, 21). However, in food processing environments, the room occupants are not just the human residents, but also the ingredients and raw materials used in processing. In this study, samples collected from the feces and hides of livestock entering the facility (animal), soil from outside the facility (soil), and the hands of facility employees (human) were collected to evaluate the impact of the potential sources on the facility microbiome. A Bayesian source-tracking analysis was used to determine the proportion of drain microbes contributed from the potential sources (22). The livestock and external facility soils were the primary sources of the microbes which established in live animal spaces. These were significant contributors to the communities within harvest spaces as well, although human sources were also major contributors. The human sources were the primary contributors to the fabrication and processing and product-holding spaces. Finally, the human and soil sources were the primary contributors to the non-product spaces (Fig. 2A). These findings align with the expected outcomes of the analysis, as the sources of microbes were the main occupants of the given spaces. There was also a proportion of the drain communities which was contributed by unknown sources. These are likely other raw materials or ingredients that were not sampled during this experiment, which would align with previous work (13).

After the microbes are introduced to the environment, the environmental conditions of the room are associated with the survival and dominance of specific organisms as part of the community. The microbial communities separated in principal coordinates by the room-function group due to the influence of specific microbes (Fig. 2B and C). The microbiome in live animal and harvest room drains were dominated by Firmicutes, which were likely deposited from the animal sources as they were highly abundant in the source communities that contributed to these spaces (Fig. S3). These spaces separate in principal coordinates due to the presence of *Moraxellaceae* and *Janthinobacteria*. The fabrication and processing spaces were consistently dominated by Pseudomonas. Indeed, the clustering of these samples was driven by three ASVs associated with Pseudomonas species (Fig. 2C). Non-product spaces that are only occupied by humans (hallways, storage rooms) are associated with an abundance of Alphaproteobacteria. These organisms, specifically orders Rhizobiales, Rickettsiales, and Sphingomonadales, have been previously reported on surfaces in food processing environments (14, 16).

Environmental conditions, especially temperature, are likely the driving factors for the differences in communities across room function. Rooms are kept at different temperatures based on their primary function, with the product holding spaces kept the coldest (below –18°C or 4°C), the fabrication and processing spaces also kept cold (below 10°C), and the live animal, harvest, and non-product spaces not temperature controlled. These uncontrolled spaces are generally room temperature or slightly colder due to the cooler activity in adjacent rooms, but during activity they may become quite warm due to body heat and hot water use. Microbial communities in built environments are strongly influenced by temperature, so this likely plays a role in the drain community assembly (1, 8, 20, 23). Specifically, the communities in cold areas (fabrication and processing, product holding) were dominated by Pseudomonas, a group of psychotropic organisms that could thrive and out-compete other organisms in these spaces. Similarly, the dominant organisms in the warm rooms tend to thrive at greater temperatures. In fact, some organisms, such as *Clostridia*, do not enter a vegetative state until the temperatures are sufficiently warm. These associations make it highly likely that the temperature (along with other environmental drivers such as relative humidity, which were not measured as part of this experiment) of the spaces, controlled due to the function of the space, influences the community assembly.

The frequency of cleaning and sanitation within the facility also influences the ability of microorganisms to form resident communities; it lowers nutritional availability, disrupts the formation of biofilms, and may force the organisms to remain in the lag growth phase, slowing the overall growth of organisms. These environmental stressors are also influenced by the function of the space. The live animal and harvest rooms, although regularly cleaned and sanitized, are still subjected to the high-volume input of potential nutrients through livestock depositions, which can introduce fecal material in both room types, and blood and viscera in harvest spaces. Conversely, the fabrication and processing spaces generally contain sanitized meat products and regularly cleaned equipment, so the introduction of nutrients is less frequent. This further elucidates the competitive advantage of organisms such as Pseudomonas in these spaces, as these organisms have a high tolerance for low nutrients and sanitizers (19).

### Development of a consistent facility microbiome.

**FIG 3:**
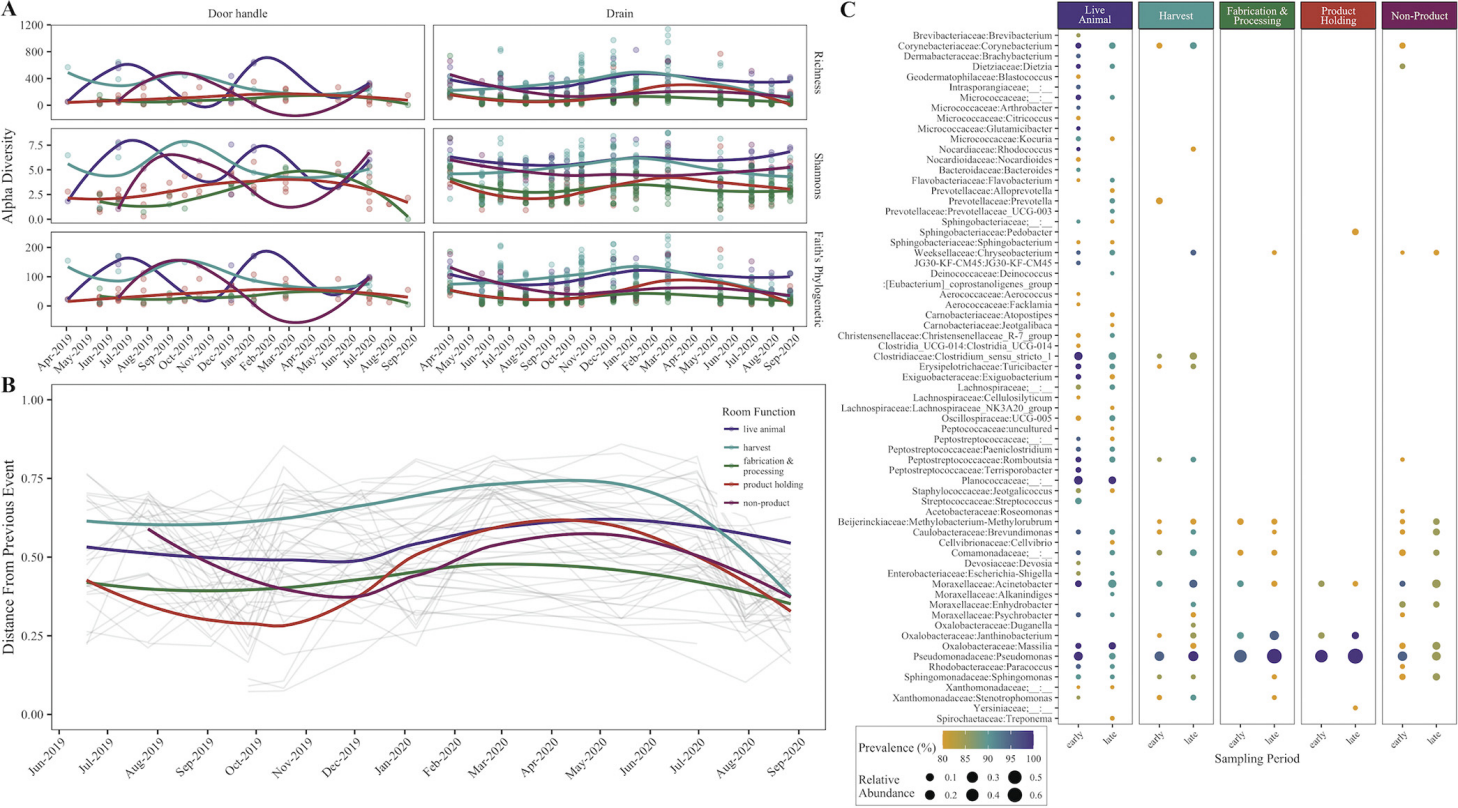
Microbial communities associated with the meat processing facility. (A) Alpha diversity changes within drains and door handles over time by facility room function. (B) Changes in similarity between microbial communities sampled from the same facility drain over time. Each point represents the change in diversity between that sampling time point and the previous time point within the same drain. A downward slope represents a trend toward a stable community, while a distance of 0 would indicate no changes in a community between sample time points. (C) Bubble plot representing the core microbes within a room-function group. Samples were grouped into two time points: the first 10 sampling points (January 2019 to February 2020) and the last 4 sampling points (March to August 2020), which corresponded to the two different time periods during which the communities approached stability.

A facility-associated microbial community forms within drains after the start of production, and potentially becomes more stable with consistent facility use. The microbial diversity within facility drains stabilizes quickly after the start of production (Fig. 3A). The mean microbial diversity within all facility drains was high at the first sampling time point, taken immediately after the post-construction clean (richness = 286, Shannon’s = 5.42, Faith’s = 87.9); it then decreased after the start of production, measured in the second sampling time point (*P* < 0.05; richness = 95.3, Shannon’s = 2.99, Faith’s = 35.0). After this, there were no significant changes in microbial richness or Shannon’s diversity between subsequent time points (*P* > 0.05), though there was still numerical fluctuation. There was, however, a difference (*P* < 0.05) in Faith’s phylogenetic diversity between time points two and three and points three and four, after which there was no further change, suggesting that the surface microbiome community diversity may have stabilized. The observation of a consistent alpha diversity agrees with other longitudinal studies of the built environment, suggesting that the diversity of indoor microbial communities is stable once microorganisms have been introduced (7, 8). Conversely, the door handle communities were more variable throughout the experimental period, as shown by a high fluctuation in alpha diversity (Fig. 3A). Door handles have more direct contact with personnel in the facility and may be cleaned less consistently than the floors, which may prevent a consistent community from establishing. This is similar to results reported by Ross and Neufeld (6) in a study of the microbiomes of door handles on a college campus, where they demonstrated that individual door handles had distinct microbiomes, door handle microbial profiles were temporary, and the diversity was directly correlated with debris present on the handle. In the study, there was rarely visible debris on the door handles sampled, but this does not exclude the possibility of the presence of contaminants. Moreover, there was variation in the types of door handles throughout the facility, even within a single room function (i.e., push bars, levers, swinging doors), which may contribute to the variable diversity, similar to the “microbial islands” observed by Ross and Neufeld (6).

The microbial community within a drain trends toward stability over time with consistent facility use. The microbial diversity within each drain was compared longitudinally to determine how similar a community identified at a single time point was to the community within the same drain at the previous sampling time point (Fig. 3B). This calculation was performed such that a decrease in the dissimilarity between samples (i.e., a negative slope) indicated that the microbial community in the drain was becoming more similar, or more stable, over time. Overall, drains within the facility trended toward stability at two points during the experimental period: November/December of 2019 and the final sampling point in September 2020. There was an increase in dissimilarity across sampling points leading into the spring of 2020 (February to March), which was likely due to the disruption of production in the facility given the university winter break (December 2019 to January 2020), when the employees left the university, and the onset of the COVID-19 pandemic closures. In June 2020, a commercial meat processing company contracted use of the GFIC facility for harvest and fabrication activities. This led to higher levels of consistent use within the facility, which was correlated with the communities again approaching a stable diversity. This is in agreement with the results from a study of the establishment of hospital microbiomes by Lax et al. (8), in which microbial communities in a patient room did not change substantially over time with a single patient in residence, but a change in occupant quickly altered the system. Overall, we hypothesize that a stable microbiome can form with consistent facility use over extended periods of time, though further experimentation is required to confirm this result.

Even though the microbial communities fluctuated in diversity and taxonomy during this experimental period, a core microbiome was established within the facility which was consistent across sampling time points. An analysis was conducted to determine which organisms were present at a relative abundance greater than 0.1 in at least 80% samples collected from each group of rooms with the same function. These organisms were considered stable members of the core community and others were considered distributed organisms, with definitions as suggested by Shade et al. (Fig. 3C) (24). Specifically, this analysis was performed on samples grouped into two time points: the first 10 sampling points (January 2019 to February 2020) and the last 4 sampling points (March 2020 to August 2020), corresponding to the two different time periods during which the communities approached stability. Interestingly, the core microbiome was similar within each functional room type across both sampling time periods, suggesting that the core microbiome was similar regardless of activity in the facility and that differences were driven by the distributed organisms. The core microbiome was defined as microbes present throughout the facility or for all but one room type/function, including Acinetobacter, *Brevundimonas*, Comamonadaceae, Pseudomonas, and *Sphingomonas*. Within a room function, live animal spaces had the most diverse core microbiome, while fabrication and processing and product holding spaces had only a few organisms in the core communities. A common core microbiome is a standard feature of built environment microbial communities, usually containing organisms found in the dust or major occupants of the environment (25, 26). In this study, the microbes which make up the core microbiome are commonly isolated in food processing environments, and are often associated with meat spoilage (15, 18, 27–29). Interestingly, these organisms are often associated with processing surface biofilms, suggesting the mechanism that allows these core organisms to persist in the environment (18, 23, 30). Future work should determine which specific functional elements lead to this persistence and whether these persistent organisms impact the microbes present on the final meat products.

### Microbial ecology of *Listeria* spp.

The overall prevalence of *Listeria* spp. in the GFIC facility during the experimental period was relatively low. Across the 14 sampling time points, 4.6% (40 out of a total of 868 sponge samples) tested positive for *Listeria* spp., and of these, 65.0% (26 out of the 40 *Listeria*-positive samples) were positive for L. monocytogenes. Overall, more *Listeria*-positive samples were collected from live animal and harvest spaces than from other room types in the facility, although *Listeria* spp. were isolated from all room function types (Table S1). This was expected, as previous study has shown that *Listeria* spp. are more prevalent in spaces with more exposure to external environments and debris (16).

**FIG 4:**
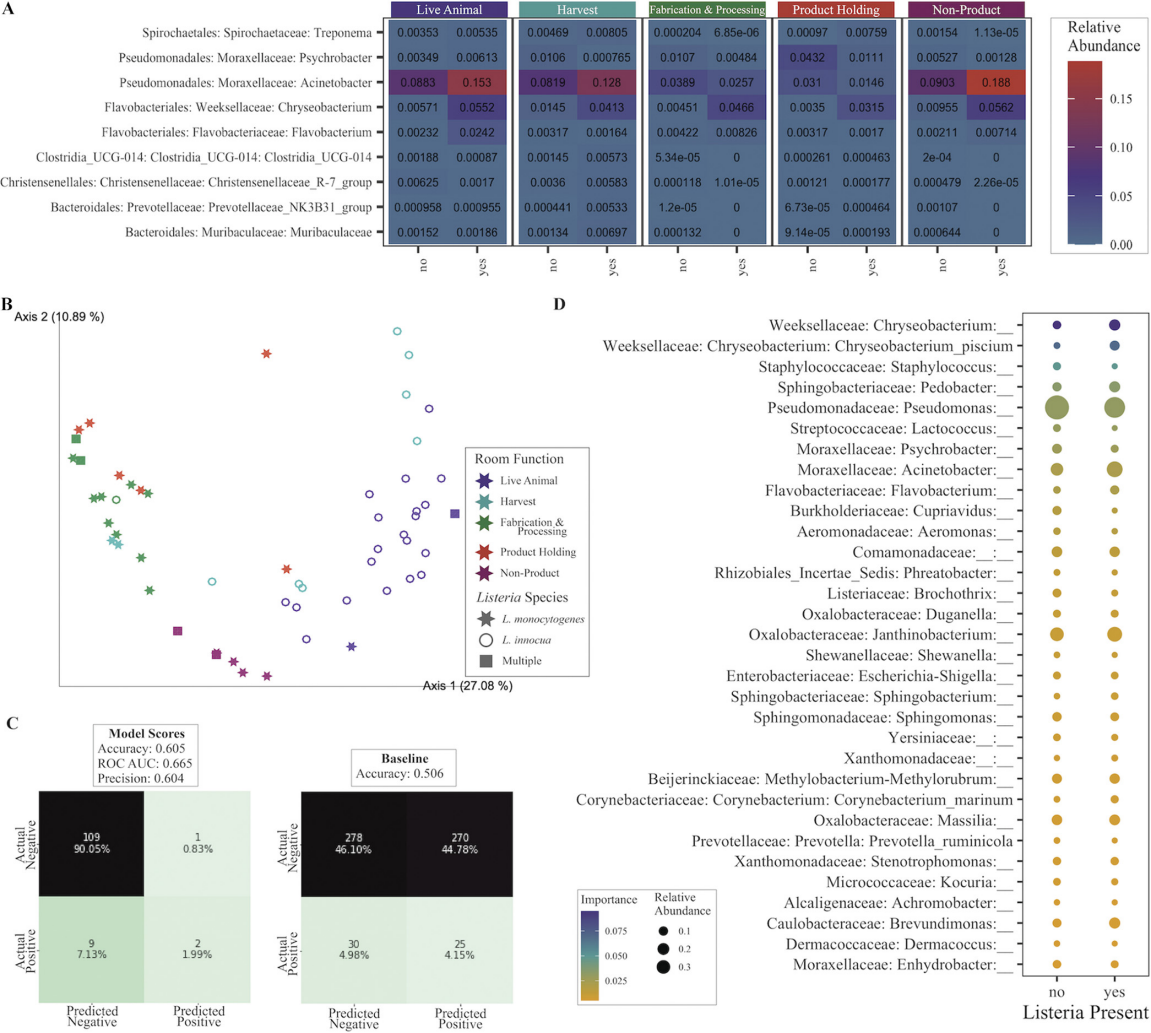
Changes to the microbial community associated with *Listeria* presence. (A) Differentially abundant taxa between *Listeria*-positive and -negative microbial communities within a room-function group. Numbers in boxes and color scale represent relative abundance. (B) Principal coordinates plot, using UniFrac distances weighted at 50%, of the microbial communities of samples which were positive for *Listeria* spp. (C) (Left) Confusion matrix and accuracy results of a Random Forest model to determine whether the microbial community can be used to identify the presence of *Listeria* in the community. (Right) Confusion matrix generated as a baseline accuracy given randomized sampling. (D) Important taxa to the Random Forest model and their relative abundance in the communities.

The presence of *Listeria* spp. within a sampling location was associated with differences in the microbial community. The microbial communities associated with the *Listeria*-positive samples were compared to those in *Listeria*-negative samples to identify any potential aspects of the community that may make it more likely to support *Listeria* growth and survival. Within a room-function group, there was no difference in the alpha diversity between *Listeria*-positive and *Listeria*-negative samples (*P* > 0.05; Fig. S4). This result was unexpected, as previous work has suggested that a higher community diversity may prevent *Listeria* growth and persistence (16, 31, 32). However, there were differences in beta diversity based on *Listeria* presence in most functional room groups, with *Listeria*-positive and -negative communities being similar only in the harvest rooms (Fig. S5). Moreover, differential abundance within a room function type revealed that *Listeria*-positive samples were associated with a higher relative abundance of *Chryseobacterium* and *Flavobacterium* in live animal rooms, *Clostridia* UCG-014, *Eubacterium*, *Muribaculaceae*, and *Prevotellaceae* in harvest rooms, and Acinetobacter, *Chryseobacterium*, and *Psychrobacter* in fabrication and processing rooms (Fig. 4A). Previous work has shown a positive correlation between *Listeria* spp. and Acinetobacter and *Chryseobacterium*, among others, in food products and food processing environments, primarily as part of a biofilm, supporting the validity of these results (17).

Given these differences associated with *Listeria*-positive and *Listeria*-negative microbial communities, it was hypothesized that the microbial communities may be predictive of *Listeria* presence in an environment. A Random Forest model with class weights was trained to conduct these predictions. The model accuracy was 0.605, which is better than random guess (0.506; Fig. 4C). This model shows promise as a potential tool for estimating *Listeria* presence without direct testing. These results are limited by the low prevalence of *Listeria* in this study; therefore, further study with more *Listeria*-positive samples may allow for increased accuracy. Previous work has used machine learning to predict the presence of *Listeria* on poultry farms with greater success, which implies that future work could generate more accurate models (33). In the current study, the important features for distinguishing these classes in the model were similar to those which were differentially abundant, including *Chryseobacterium*, *Psychrobacter*, Acinetobacter, and *Flavobacterium* (Fig. 4D). Moreover, although organisms in the Listeriaceae family are important features, the genus *Listeria* itself is not important to the model. The *Listeria* genus was only detected via 16S rRNA gene sequencing in 28 of the total samples, and only one of these was also found to be *Listeria*-positive from the GENE-UP PCR analysis. This suggests that amplicon sequencing methodologies do not recover *Listeria* as well as PCR for these sample types, but other signals in the microbial community may be sufficient to represent its presence.

Interestingly, the presence of different *Listeria* species was stratified by room function, similar to the environmental separation seen in the general microbial communities. *Listeria*-positive isolates were identified to the species level following confirmation, and different species were correlated with specific microbiomes, which were separated based on room function (Fig. 4B). Listeria innocua-positive samples were associated with live animal and harvest rooms, while L. monocytogenes-positive samples were associated with fabrication and processing, product holding, and non-product spaces. It is unclear whether these spatial patterns of *Listeria* species are driven by the microbiome present in the drain from which *Listeria* was isolated, or whether the *Listeria* species is driven by the same environmental conditions that likely drive the overall microbial ecology. Previous work has shown the two species are impacted differently by environmental conditions; L. monocytogenes survives at lower temperatures, has greater acid and antimicrobial resistance, and is more metabolically flexible than L. innocua, all of which may adapt L. monocytogenes to better survive in the colder and less nutrient-rich fabrication and processing rooms. Therefore, it appears the same environmental conditions which shape the microbial communities could be driving the spatial differentiation of *Listeria* species in the environment (30–32). This work represents an exciting new finding in the stratification of these important pathogens within meat processing facilities.

### Conclusions.

The objectives of this study were to determine whether a stable microbial community develops in a small meat processing facility, to determine how environmental factors and room function drive community formation, and to elucidate the relationship between microbial communities and the presence of *Listeria* spp. To address this, the microbial communities and *Listeria* presence in drains and on door handles in a meat processing facility were monitored for the first 18 months of operation. From these observations, it is clear that a core microbiome forms quickly in meat processing facilities, and the distributed organisms not part of the core community become more stable with more consistent facility use. The communities assemble differently due to environmental conditions; different sources contribute microbes to distinct spaces, and then the conditions in a facility room, especially temperature, shape and maintain the communities. *Listeria* spp. presence is associated with a higher relative abundance of a few specific organisms, including *Chryseobacterium*, Acinetobacter, and *Flavobacterium*. These differences have the potential to lead to predictive models to determine the presence of *Listeria* spp. in a microbial community without direct sampling. Overall, these results have major implications for the meat processing industry, as it demonstrates how microbial communities form and persist in meat processing environments, and their potential to harbor important pathogens, including L. monocytogenes.

## MATERIALS AND METHODS

### The Global Food Innovation Center.

The Global Food Innovation Center is a state-of-the-art-food processing and research center associated with the Department of Animal Sciences at Colorado State University (Fort Collins, Colorado). The facility is 36,000 ft^2^, with 20,000 ft^2^ directly allocated to meat laboratory spaces. Spaces include live animal handling and overnighting holding facilities, harvest spaces, fabrication (the breakdown of carcasses to primals and cuts) spaces, processing rooms, smokehouses, ready-to-eat spaces separated from previous rooms by pass-through smokehouses, and several carcass coolers and product holding rooms (see facility map in Fig. 1A). The facilities are designed to operate similarly to a small commercial facility (Fig. 1C). During normal production, live animals are introduced in the livestock holding space. Then, animals are harvested and converted to carcasses in the hide-on harvest and hide-off harvest rooms. The carcass chill cooler is used to rapidly reduce carcass temperatures immediately after harvest; then, the carcass holding cooler is used to store carcasses until fabrication. The carcasses are converted to saleable products in the fabrication and processing spaces. If a product is to be sold fresh (unprocessed/not cooked), it is moved to the product cooler or freezer immediately after fabrication. If it is further processed, it is cooked in the smokehouses between cooked meats and the in-process cooler, then finished in the cooked meats packaging room before being stored in the product cooler or freezer.

Room temperatures are carefully managed to maintain the cold chain during production. Carcass and product coolers (carcass chill cooler, carcass holding, product cooler, in-process cooler) are kept below 4°C, the product freezer is kept below –18°C, and processing rooms (fabrication, processing, cooked meats packaging) are kept below 10°C. The temperatures of other spaces are not precisely controlled because they do not contain products susceptible to spoilage or contamination. Construction of the facility began in December 2017 and was completed in January 2019. Production began in the facility on January 12, 2019. Production was paused from February to May 2020 as a consequence of COVID-19 restrictions. Additionally, beginning in June 2020, a commercial meat processing company began to operate in the GFIC facility due to loss of the company facilities, which increased and altered the production rates and personnel present in the facility during this period.

### Experimental design and sample collection.

A nested longitudinal study design was used to capture the origins and changes in microbial communities in the newly constructed meat processing facility. Samples were collected approximately monthly from drains (*n *=* *630) and door handles (*n *=* *300) in production, storage, and non-product spaces in the GFIC facility. The first sampling event occurred immediately after the post-construction clean but before production began within the facility. This point was staggered because construction of different spaces was completed at different times (January to April 2019). Following the final initial sampling event, samples throughout the facility were collected approximately monthly until August 2020, with a short cessation from February to May 2020 when the facility was closed due to COVID-19 regulations, as described above. The personnel performing sample collection wore recommended personal protective equipment (disposable coats, disposable boot covers, hair nets, hard hats, gloves) and moved from “clean” (ready-to-eat, fabrication) to “dirty” (harvest, livestock holding) spaces to reduce the amount of contamination transferred through the facility and to follow facility regulations.

Samples were collected from drains and door handles throughout the GFIC facility (Fig. 1A). These sampling points were chosen for several reasons. The goal of the research project was to capture the microbiome associated with the processing facility, and these points were representative of the facility itself. These are also permanent fixtures, unlike equipment and food processing surfaces, and could be resampled reliably throughout the experimental period. These are also key sites to represent potential contamination and persistence of *Listeria* spp., since drains especially are known to be reservoirs for *Listeria* in processing facilities (34, 35). Finally, given regulatory restrictions at the facility, the potential to find L. monocytogenes on food contact surfaces was too great a risk to negotiate with facility management. At each sampling point, a sterile double-headed SWUBE swab (BD; Franklin Lakes, NJ) was used to collect a sample for microbiome analysis, and an EZ Reach sponge pre-moistened with HiCap neutralizing broth (World Bioproducts, Bothell, WA) was used to collect a sample for detection of *Listeria* spp. (Whirl-Pak, Madison, WI). Drain samples were collected by swabbing both the top and bottom of the drain cover and the opening to the drainpipe. The facility contains several types of door handles which had to be swabbed differently; however, in general, samples were collected by swabbing the part of the handle with human hand contact and the surface an employee would push to open the door. If a sampling point had two doors, the right-side door handle was chosen to be swabbed. The smokehouse doors had two handles, one to open the door and one to open a viewing window; at this site, both of these handles were swabbed as one sample. After collection, swabs for microbiome analysis were immediately placed on ice, then frozen at –4°C after completion of a sampling event to be stored until sequencing. Sponge samples collected for *Listeria* spp. analysis were processed immediately following sample collection, as described below.

To identify potential sources of microbes found within the facility, samples were taken from employee skin, animals being introduced to the facility, and the surrounding environment using a double-headed SWUBE swab (BD, Franklin Lakes, NJ). Human skin samples were taken by providing employees with a swab and instructing them to vigorously swab their dominant hand. Animal samples were collected at the time of harvest using the sterile SWUBE swabs. Skin or hide swabs were collected from the left shoulder of the animal immediately after the exsanguination process was initiated, fecal samples were collected from the rectum before sealing of the caudal end of the gastrointestinal system, a pre-wash carcass sample was collected from the left shoulder after evisceration but before final trim, and the post-chill carcass sample was collected from the left shoulder after the carcass had undergone 24 h of chilling. Environmental soil samples were collected from roads and sidewalks leading into the main facility doors.

Animals included in the experiment to collect potential microbial source samples were harvested as part of normal facility operation. Animal use was reviewed by the CSU Institutional Animal Care and Use Committee and declared to be exempt with IACUC waiver no. 2019-102-ANSCI. Human subjects used for hand swabs as potential microbial source samples provided informed consent and samples were de-identified. Protocols were approved by the CSU Institutional Review Board under protocol no. 18-8414H. The handling of Listeria monocytogenes was approved by the CSU Biosafety Committee as project 19-001B.

### DNA extraction and sequencing.

Microbial communities were characterized using paired-end 16S rRNA gene sequencing, as demonstrated in Fig. 1B. DNA was extracted from the sampling swabs using the Qiagen PowerSoil kit (Qiagen; Hilden, Germany) following the manufacturer’s recommendations. In order to collect adequate DNA for sequencing from the door handle samples, DNA was extracted from both heads of the swab, with only one head used for the drain samples and the other retained in case re-sequencing was needed. Extraction was conducted using 96-well plates, with seven negative controls and one mock community-positive control (Zymo; Irving, CA) per plate.

After extraction, DNA was amplified and sequenced following EMP Protocols using the 515f/806r primers (www.earthmicrobiome.org) (36). PCR primers included error-correcting Golay barcodes to allow for multiplexing. PCR products were quantified using the Picogreen Quant-iT (Invitrogen, Life Technologies; Grand Island, NY) and then pooled at equimolar concentrations for sequencing. Pools were sequenced using a 500-cycle kit on the Illumina miSeq sequencing platform (Illumina; San Diego, CA). Due to the high number of samples and the long time period across which samples were collected, samples were sequenced across four sequencing lanes, randomized across plates so that no one run contained samples from all sampling events, room, or sample type to prevent confounding by technical artifacts.

### *Listeria* prevalence.

Immediately after collection, a 100-mL volume of LPT broth (bioMérieux; Marcy-l’Etoile, France) was added to each sponge sample bag. Sponges were hand massaged for 60 s through the outside of the bag and then incubated at 37°C for 22 h. Following the enrichment step, samples were tested for the presence of *Listeria* spp. using the GENE-UP real-time PCR pathogen detection system (bioMérieux). The samples were prepared for GENE-UP PCR following the manufacturer’s instructions. Briefly, 20 μL of the incubated sample was added into individual lysis tubes and placed into a 96-well plate. Ten μL of the lysated samples was placed into the provided PCR mixture and subjected to PCR amplification in the GENE-UP thermocycler. Two positive and two negative controls were used, with the positive controls being pure L. innocua and L. monocytogenes cultures, respectively. Negative controls were uninoculated LPT broth. Results were automatically determined within the instrument following run time (~1-h cycling time). Samples determined as positive were subjected to culture confirmation with microbiological plating methods using Modified Oxford plates supplemented with yeast extract with an incubation time of 48 h at 37°C (37). An additional plating step was performed if samples exhibited typical *Listeria* spp. growth. Positive samples were then subjected to a second GENE-UP PCR run for PCR confirmation.

### *Listeria* spp. identification.

Confirmed-positive isolates were subjected to species-level identification utilizing the API *Listeria* kit (bioMérieux) following the manufacturer’s instructions. Briefly, frozen stock cultures of confirmed *Listeria* spp. were placed onto polymyxin acriflavin lithium-chloride ceftazidime esculin mannitol (PALCAM) agar and incubated for 48 h at 37°C; a purification step was then performed on these cultures. Next, one isolated colony from the purified plates was individually plated onto PALCAM agar and incubated at 37°C for 24 h. Two to three well-isolated colonies from each sample were individually placed into the API Suspension Medium, with turbidity equivalent to 1 McFarland. Once the inoculum was prepared, each sample was then pipetted into the wells (100 μL for the DIM [Differentiation/Innocua/Monocytogenes] test, an enzymatic substrate used for differentiation of *L. innocua* and L. monocytogenes, and 50 μL for the remainder of the tests in the test strip). All incubation boxes were closed and incubated at 37°C for roughly 24 h. Following incubation of the strips, ZYM B reagent (provided by the manufacturer) was then placed into the DIM test, and the reactions from all wells were then read and recorded. A four-digit numerical profile was obtained from the results of the tests in the kit. The numerical profile was then entered into the APIWEB software database, and species-level identification of the sample was attained.

### Microbial community analysis.

After sequencing, data were demultiplexed and de-noised with DADA2 using QIIME2 version 2020.8 software (38, 39). Taxonomy was classified using the SILVA 138 99% database with the QIIME2 feature-classifier plugin, which classifies reads using a pre-trained machine learning classifier (40, 41). The taxonomy was used to filter out reads classified as chloroplast and mitochondria, as these sequences were not considered part of the true microbiome. Additional filtering steps were used to remove sequences which appeared in less than 10% of samples.

To conduct phylogenetic diversity analyses, a phylogenetic insertion tree was constructed using the SEPP program with the SILVA 138 tree as a backbone (42, 43). Next, data were rarefied to 9,204 ASVs/sample and a phylogenetic diversity analysis was conducted using the core metrics pipeline in QIIME2 (39). Alpha diversity statistical comparisons were made using a Kruskal-Wallis test with a Benjamini-Hockberg multiple-testing correction (44). Beta diversity was analyzed using a generalized UniFrac test with a weight of 50%, and statistical comparisons were made using a permutational multivariant analysis of variance (PERMANOVA) test with multiple-testing correction (45, 46). Additionally, community differences were visualized using the DEICODE pipeline to generate a Robust Aitchison Principal Components Analysis (47). Changes in community diversity over time were analyzed using the QIIME2 longitudinal plugin (48). The first distances method with the generalized UniFrac metric was used to calculate differences in beta diversity between each sampling event to demonstrate the movement of a microbial community within a single drain towards a stable community over time. This calculation was visualized using a volatility plot with changes summarized across room function and evaluated statistically using a linear mixed-effects model, with the first distance as the dependent variable, room function and time as fixed effects, and drain ID as a random effect. A negative slope was used to indicate a trend toward stability in the community.

Taxonomy changes across time and space in the facility were also evaluated. Organisms which changed significantly within a room-function group over time were identified using an Analysis of Composition of Microbiomes analysis (49). Organisms with a large, statistically significant change from this analysis and with biological significance based on prior knowledge of food processing microbiota were further investigated using a spatial relative abundance map generated with the SitePainter tool (50). Microbial sources were analyzed using SourceTracker2 software (22). The analysis was conducted using the developer version of the software and following developer instructions. Samples taken from facility drains were used as the sinks (locations to which microbes were transferred) and samples collected from livestock feces and hides, employee hands, and soils outside the facility were used as sources (locations from which microbes were transferred). Throughout the study, all statistical analysis was conducted with an alpha of 0.05.

### *Listeria* data analysis.

A generalized linear model was fit, and the predictions of the presence and distribution of presumptive and confirmed *Listeria* spp. throughout the facility were made using estimated marginal means. Additionally, a chi-squared Pearson’s correlation test was used to analyze the relationship between room function and *Listeria* species. The microbial communities associated with *Listeria* presence were then analyzed to determine the role of the microbiome as a reservoir and indicator of the presence of *Listeria* spp. pathogen residence. Comparisons of alpha diversity, beta diversity, and taxonomy were conducted to evaluate the differences in communities between *Listeria*-positive and *Listeria*-negative samples using the statistical methods described above. Machine learning models were constructed to determine whether the microbial community was predictive of the presence of *Listeria* spp. in a facility drain. Microbial community data in biom format were imported into Python using the Calour package (51). Then, models were trained using a Random Forest classifier from scikit learn version 0.22.1 with nested k-fold cross validation (41). Relative abundances of microbial taxa were used as the predictors and *Listeria* presence was used as the response variable. The class weight parameter was used to artificially increase the weight of *Listeria*-positive samples to balance the data set. Model accuracy was assessed during cross-validation using the accuracy metric. A baseline model was trained using the scikit learn dummy classifier with the uniform strategy.

### Data availability.

16S rRNA gene sequencing data supporting the conclusions of this article are available in the EBI repository, accession no. ERP130385, and on the QIITA platform under study ID 12948.
