# Supplementary material for: A Pilot Study: the Development of a Facility-Associated Microbiome and Its Association with the Presence of Listeria Spp. in One Small Meat Processing Facility
Source: Microbiol Spectr. 2022 Aug 18;10(5):e02045-22. doi: 10.1128/spectrum.02045-22 (PMC9603805; doi:10.1128/spectrum.02045-22)
Supplement: Supplemental file 1 — Supplemental material. Download spectrum.02045-22-s0001.pdf, PDF file, 4.5 MB [file spectrum.02045-22-s0001.pdf]

SI Figure 1. Comparison of the relative abundance of taxa found in positive control samples sequenced on Illumina plates and the expected community composition (zymo). The numbers associated with samples represent the extraction plate and sequencing run with which each control was associated.

SI Figure 2. The spatial movement of microorganisms through drains in the meat processing facility. Darker colors represent a higher relative abundance of the given organism in the drain associated with that position in the facility.

SI Figure 3. Taxonomy of potential microbial sources compared to the functional room type.

SI Figure 4. The alpha diversity of microbial communities exhibiting the presence or absence of *Listeria* was analyzed based on room function.

SI Figure 5. PERMANOVA results comparing microbial communities that are *Listeria*-positive and -negative within a functional room group. Differences indicate that the communities are dissimilar based on the presence of *Listeria* spp.

SI Table 1. The number of confirmed *Listeria*-positive samples collected in each room function group at each timepoint. Numbers are segregated by each species.

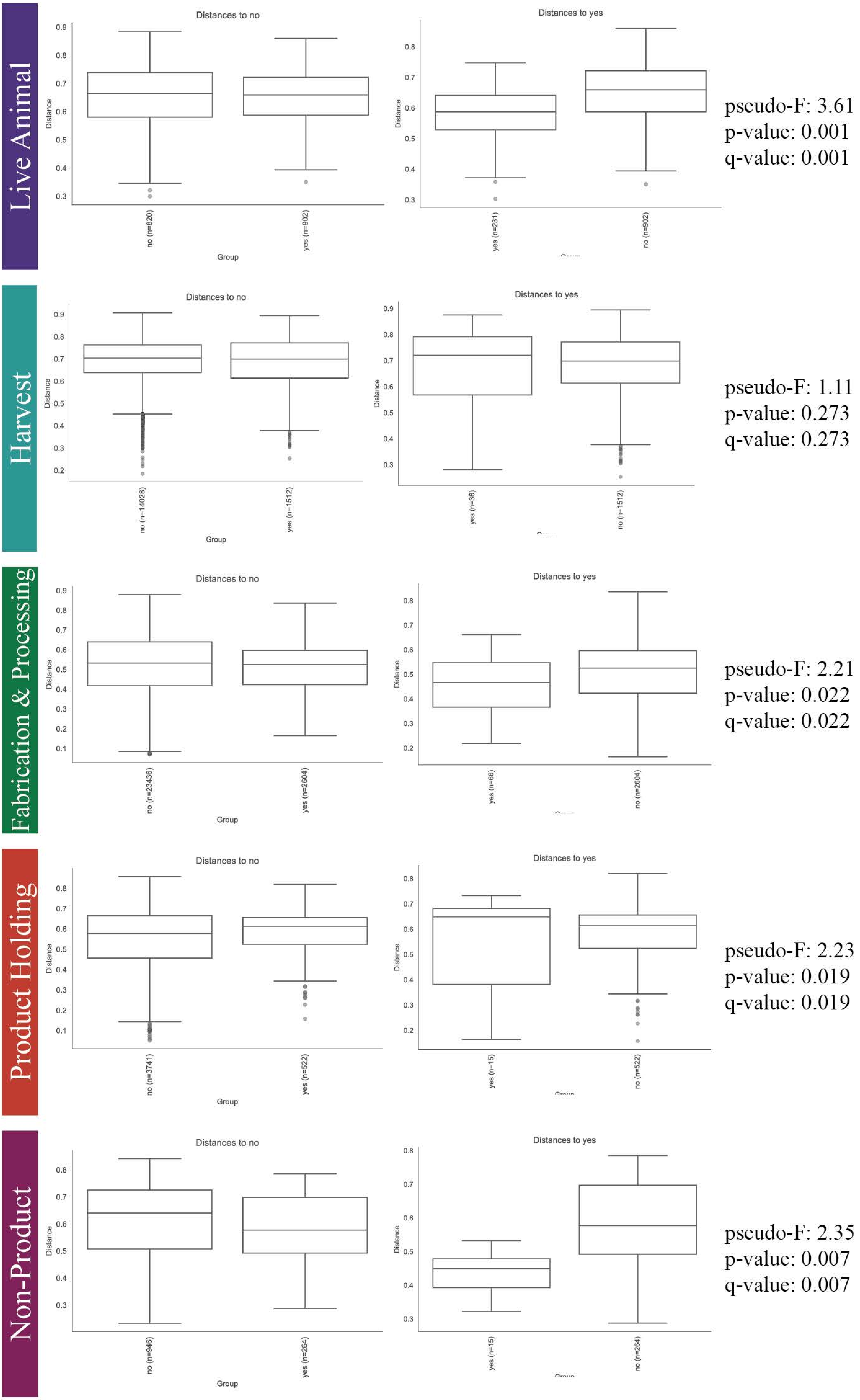
Live Animal
Distances to no
Distances to yes
Distance
Group
no (n=820)
yes (n=902)
yes (n=231)
no (n=902)
pseudo-F: 3.61
p-value: 0.001
q-value: 0.001
Harvest
Distances to no
Distances to yes
no (n=14028)
yes (n=1512)
yes (n=36)
no (n=1512)
pseudo-F: 1.11
p-value: 0.273
q-value: 0.273
Fabrication & Processing
Distances to no
Distances to yes
no (n=23436)
yes (n=2604)
yes (n=66)
no (n=2604)
pseudo-F: 2.21
p-value: 0.022
q-value: 0.022
Product Holding
Distances to no
Distances to yes
no (n=3741)
yes (n=522)
yes (n=15)
no (n=522)
pseudo-F: 2.23
p-value: 0.019
q-value: 0.019
Non-Product
Distances to no
Distances to yes
no (n=946)
yes (n=264)
yes (n=15)
no (n=264)
pseudo-F: 2.35
p-value: 0.007
q-value: 0.007

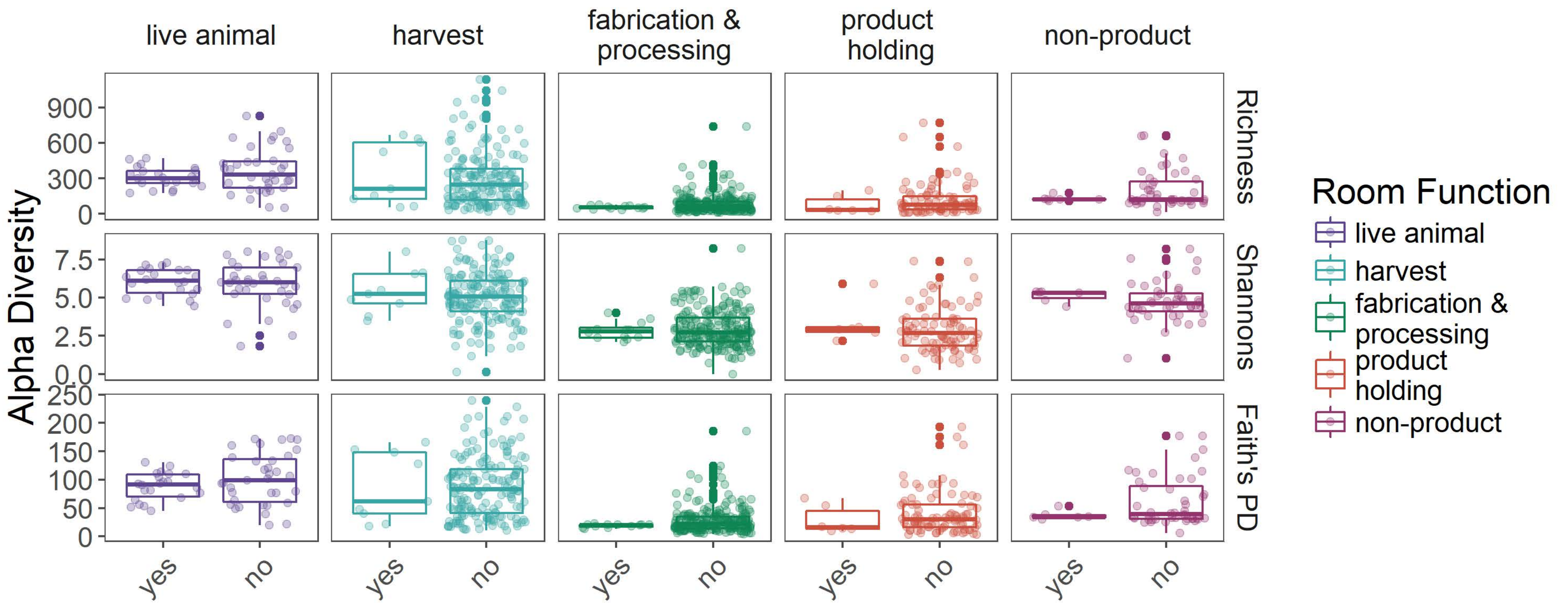

live animal
harvest
fabrication & processing
product holding
non-product
Richness
Shannons
Faith's PD
Alpha Diversity
900
600
300
0
7.5
5.0
2.5
0.0
250
200
150
100
50
0
yes
no
Room Function
live animal
harvest
fabrication & processing
product holding
non-product

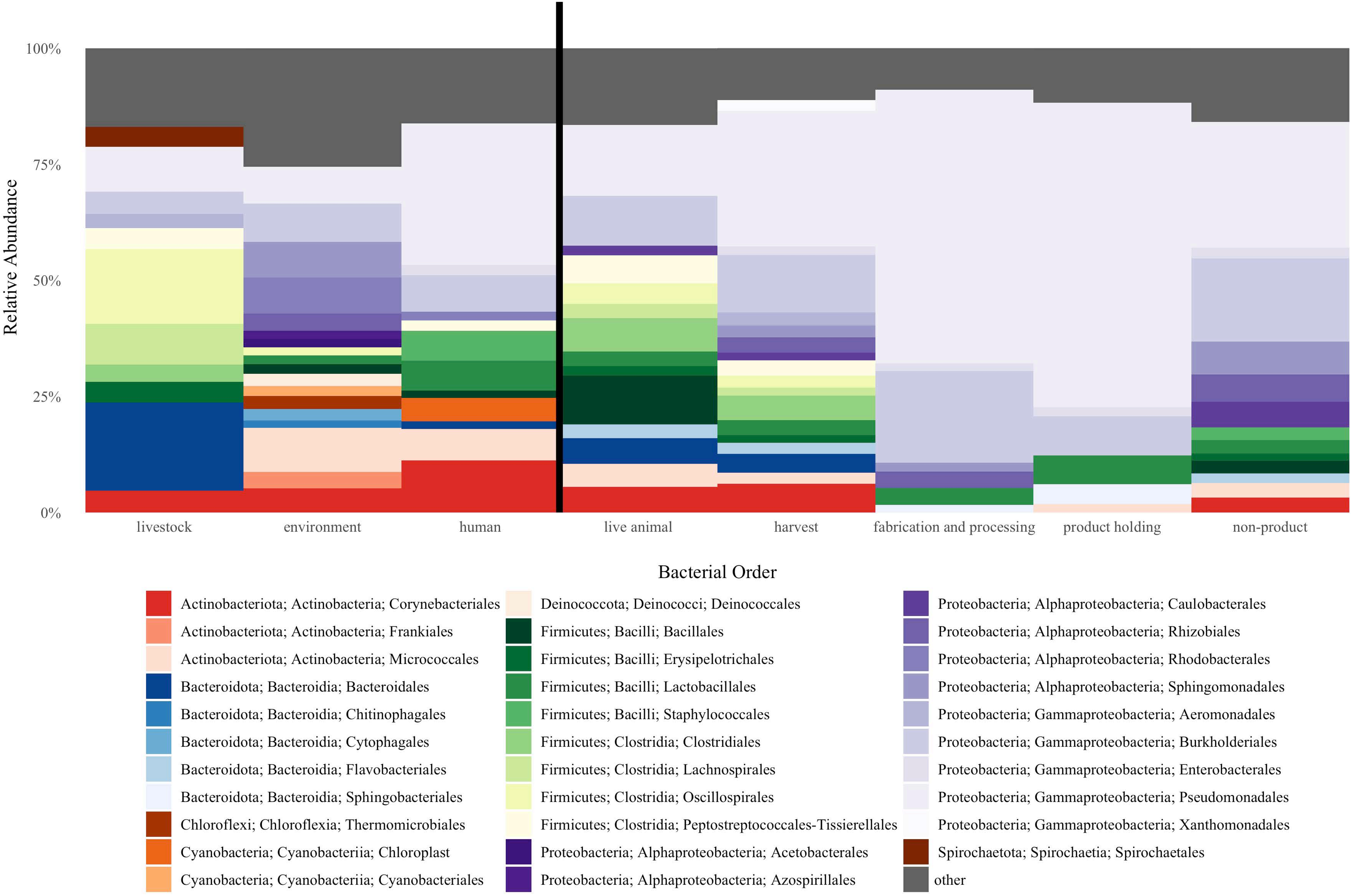

Relative Abundance
100%
75%
50%
25%
0%
livestock
environment
human
live animal
harvest
fabrication and processing
product holding
non-product
Bacterial Order
Actinobacteriota; Actinobacteria; Corynebacteriales
Actinobacteriota; Actinobacteria; Frankiales
Actinobacteriota; Actinobacteria; Micrococcales
Bacteroidota; Bacteroidia; Bacteroidales
Bacteroidota; Bacteroidia; Chitinophagales
Bacteroidota; Bacteroidia; Cytophagales
Bacteroidota; Bacteroidia; Flavobacteriales
Bacteroidota; Bacteroidia; Sphingobacteriales
Chloroflexi; Chloroflexia; Thermomicrobiales
Cyanobacteria; Cyanobacteriia; Chloroplast
Cyanobacteria; Cyanobacteriia; Cyanobacteriales
Deinococcota; Deinococci; Deinococcales
Firmicutes; Bacilli; Bacillales
Firmicutes; Bacilli; Erysipelotrichales
Firmicutes; Bacilli; Lactobacillales
Firmicutes; Bacilli; Staphylococcales
Firmicutes; Clostridia; Clostridiales
Firmicutes; Clostridia; Lachnospirales
Firmicutes; Clostridia; Oscillospirales
Firmicutes; Clostridia; Peptostreptococcales-Tissierellales
Proteobacteria; Alphaproteobacteria; Acetobacterales
Proteobacteria; Alphaproteobacteria; Azospirillales
Proteobacteria; Alphaproteobacteria; Caulobacterales
Proteobacteria; Alphaproteobacteria; Rhizobiales
Proteobacteria; Alphaproteobacteria; Rhodobacterales
Proteobacteria; Alphaproteobacteria; Sphingomonadales
Proteobacteria; Gammaproteobacteria; Aeromonadales
Proteobacteria; Gammaproteobacteria; Burkholderiales
Proteobacteria; Gammaproteobacteria; Enterobacterales
Proteobacteria; Gammaproteobacteria; Pseudomonadales
Proteobacteria; Gammaproteobacteria; Xanthomonadales
Spirochaetota; Spirochaetia; Spirochaetales
other

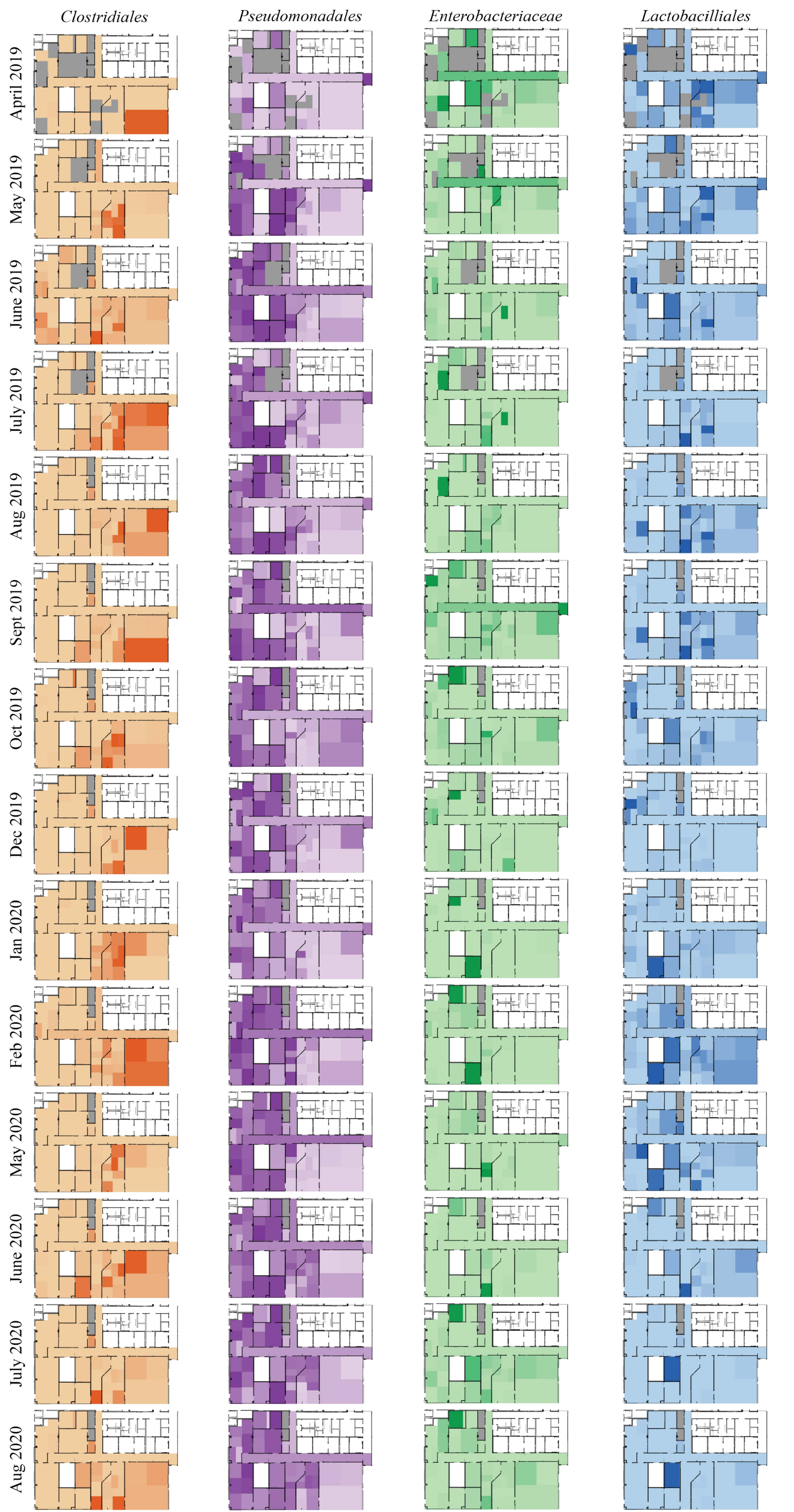
Clostridiales
Pseudomonadales
Enterobacteriaceae
Lactobacilliales
April 2019
May 2019
June 2019
July 2019
Aug 2019
Sept 2019
Oct 2019
Dec 2019
Jan 2020
Feb 2020
May 2020
June 2020
July 2020
Aug 2020

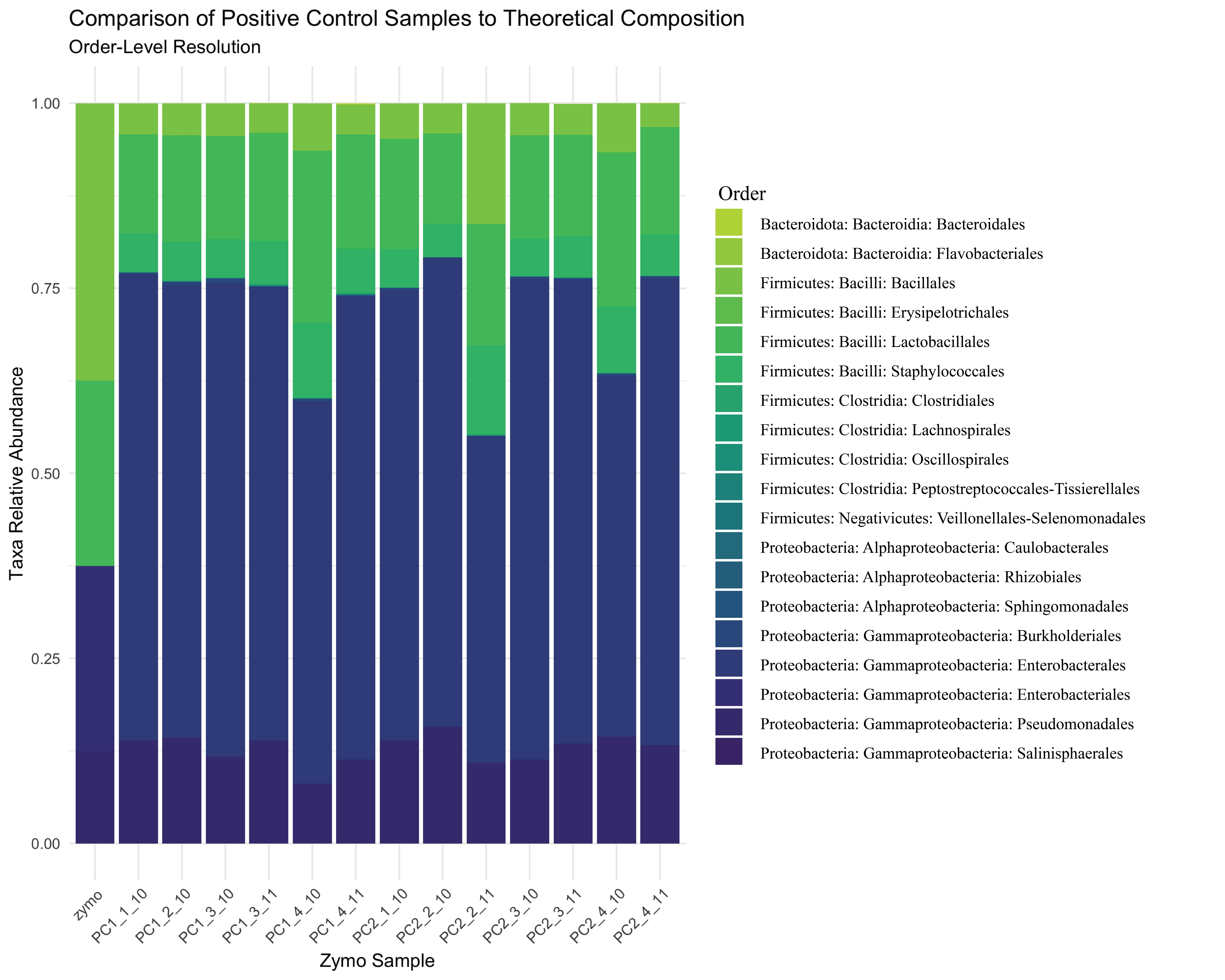

Comparison of Positive Control Samples to Theoretical Composition
Order-Level Resolution
Taxa Relative Abundance
1.00
0.75
0.50
0.25
0.00
zymo
PC1_1_10
PC1_2_10
PC1_3_10
PC1_3_11
PC1_4_10
PC1_4_11
PC2_1_10
PC2_2_10
PC2_2_11
PC2_3_10
PC2_3_11
PC2_4_10
PC2_4_11
Zymo Sample
Order
Bacteroidota: Bacteroidia: Bacteroidales
Bacteroidota: Bacteroidia: Flavobacteriales
Firmicutes: Bacilli: Bacillales
Firmicutes: Bacilli: Erysipelotrichales
Firmicutes: Bacilli: Lactobacillales
Firmicutes: Bacilli: Staphylococcales
Firmicutes: Clostridia: Clostridiales
Firmicutes: Clostridia: Lachnospirales
Firmicutes: Clostridia: Oscillospirales
Firmicutes: Clostridia: Peptostreptococcales-Tissierellales
Firmicutes: Negativicutes: Veillonellales-Selenomonadales
Proteobacteria: Alphaproteobacteria: Caulobacterales
Proteobacteria: Alphaproteobacteria: Rhizobiales
Proteobacteria: Alphaproteobacteria: Sphingomonadales
Proteobacteria: Gammaproteobacteria: Burkholderiales
Proteobacteria: Gammaproteobacteria: Enterobacterales
Proteobacteria: Gammaproteobacteria: Enterobacteriales
Proteobacteria: Gammaproteobacteria: Pseudomonadales
Proteobacteria: Gammaproteobacteria: Salinisphaerales

	**Product Holding**		**Fabrication & Processing**		**Harvest**		**Live Animal**		**Non-Product**	
	L. mono	*L. innocua*	*L. mono*	*L. innocua*	*L. mono*	*L. innocua*	*L. mono*	*L. innocua*	*L. mono*	*L. innocua*
**4/3/2019**	0	0	0	0	0	0	0	0	0	0
**5/21/2019**	0	1	0	0	0	0	0	0	0	0
**6/18/2019**	0	0	0	0	0	0	0	1	0	0
**7/26/2019**	0	0	0	0	0	0	0	0	0	0
**9/3/2019**	0	0	0	0	0	0	0	0	0	0
**9/26/2019**	0	0	1	0	0	0	0	0	0	0
**10/18/2019**	0	0	0	0	0	5	0	3	0	0
**12/5/2019**	0	0	0	0	0	0	0	0	0	0
**1/8/2020**	0	0	0	0	0	0	0	0	0	0
**2/25/2020**	0	0	0	0	0	0	0	0	0	0
**5/14/2020**	0	0	0	0	0	0	0	0	0	0
**6/30/2020**	0	0	0	0	0	2	0	3	0	0
**7/30/2020**	0	0	0	1	0	0	0	3	0	0
**8/27/2020**	6	0	6	0	1	0	0	2	3	0
**TOTAL**	6	1	7	1	1	7	2	12	3	0
